# Innovations in e-health

**DOI:** 10.1007/s11136-013-0458-x

**Published:** 2013-07-14

**Authors:** Paul Wicks, Jon Stamford, Martha A. Grootenhuis, Lotte Haverman, Sara Ahmed

**Affiliations:** 1 PatientsLikeMe, Research and Development, Cambridge, MA USA; 2 Parkinson’s Movement, St Botolph’s, Aldgate High Street, London, EC3 1AB UK; 3 The Cure Parkinson’s Trust, St Botolph’s, Aldgate High Street, London, EC3 1AB UK; 4 Pediatric Psychosocial Department, Academic Medical Center, Emma Children’s Hospital, Amsterdam, The Netherlands; 5 Faculty of Medicine, School of Physical and Occupational Therapy, McGill University, McGill University Health Center, Centre de recherche interdisciplinaire en réadaptation (CRIR), 3654 Prom Sir-William-Osler, Montreal, QC H3G 1Y5 Canada

**Keywords:** E-health, Social network, Internet, Clinical practice, Patient-reported outcome

## Abstract

The theme of ISOQOL’s 19th Annual Conference in Budapest, Hungary, was *The Journey of Quality of Life Research: A Path Towards Personalized Medicine. Innovations in e*-*health* was one of four plenary panels. E-health is changing the landscape of clinical practice and health care, but the best way to leverage the many promised benefits of emerging e-health technologies is still not clear. The Innovations in e-health panel presented emerging changes in technologies and applications that will facilitate clinical decision making, improve quality and efficiency of care, engage individuals in clinical decision making, and empower them to adopt healthy behaviors. The purpose of this paper was to present emerging trends in e-health and considerations for successful adoption of new technologies, and an overview of each of the presentations in the e-health plenary. The presentations included a personal perspective on the use of technology for self-monitoring in Parkinson’s disease, an overview of online social networks and emerging technologies, and the collection of patient-reported outcomes through web-based systems in clinical practice. The common thread across all the talks was the application of e-health tools to empower individuals with chronic disease to be actively engaged in the management of their health. Considerations regarding data ownership and privacy, universal access to e-health, interactivity between different types of e-health technologies, and tailoring applications to individual needs were explored.

## Introduction

The theme of ISOQOL’s 19th Annual Conference in Budapest, Hungary, was *The Journey of Quality of Life Research: A Path Towards Personalized Medicine. Innovations in e*-*health* was one of four plenary panels. E-health is changing the landscape of health care and refers to *“an emerging field in the intersection of medical informatics, public health and business, referring to health services and information delivered or enhanced through the Internet and related technologies”*[1]. The best way to leverage emerging technologies to improve quality of life is still not clear, though pockets of implementation [2] and early successes have been promising [3]. Actively engaging clinicians and patients’ in the design and iteration of these new technologies will be a key in ensuring widespread adoption. Continuous evaluation will be needed to quantify their impact on quality of care, individuals’ health and quality of life, and the reduction in healthcare costs.

The growing fiscal and social burden of managing and preventing chronic diseases remains one of the greatest challenges facing healthcare systems worldwide [4], and e-health can help meet this challenge in three key ways. The first is increasing efficiency in health care, thereby decreasing costs. This might be through avoiding duplication of unnecessary diagnostic or therapeutic interventions, through enhanced communication that enables care to have a wider reach [5], or through off-loading of burdensome but simple tasks to technology. Improving efficiency does not need to come at the expense of the second area, enhancing the quality of health care. For example, e-health may improve quality of care by maintaining contact with patients between clinical visits, allowing comparisons between different providers, and incorporating patient reports of health and satisfaction into quality assurance for payers and commissioners, as well as other patients.

The third area of opportunity is empowering individuals to actively manage their health and to adopt healthy behaviors. e-health technologies such as personal health records provide an opportunity for (1) ongoing disease monitoring and feedback from the care team [6]; (2) providing enhanced self-management interventions and case management when problems are identified [7]; and (3) sharing of clinical information and treatment goals with the patient. In this way, it is hoped that e-health could open new avenues for patient-centered medicine, and eventually enable evidence-based patient choice.

Patients are leading changes in the way they manage their health through peer-to-peer support networks such as *PatientsLikeMe*. Online networks enable patients to share and compare and contrast different diagnoses and treatments with people who have the same conditions who are anywhere in the world. Members of the online community can ask for advice, learn from each other, discuss test results, and compare how different medications, treatments, or combinations of drugs might or might not be working [8]. This sharing of information creates a more informed and empowered patient and can lead to a radical reconfiguring of the patient/care team relationship. As health professionals are no longer the only source of information, the relationship becomes more equal and collaborative.

Along with their potential, however, e-health technologies might also carry some risks. For instance, some among the medical community express concerns regarding the quality of the information accessed online, as well as patients’ health literacy and their ability to understand such information [9]. Some studies suggest a shift of trust from physicians’ advice to online and peer-to-peer sources, especially among patients dissatisfied with their medical provider. It remains unclear what the consequences might be however; the only systematic review of harms inflicted by the Internet was conducted in 2002 and found only a single death [10–12]. Research regarding the impact of e-health and potential risks of emerging technologies is therefore scarce, and future studies will need to rigorously address questions related to potential harms of e-health.

The *Innovations in e*-*health* panel presented emerging changes in technologies and applications that will facilitate clinical decision making, improve quality and efficiency of care, engage individuals in clinical decision making, and empower them to adopt healthy behaviors. The presenters also addressed potential limitation of existing systems and risks associated with e-health applications. Particular focus was paid to applications supported by smart phones, monitoring devices, and collection of patient-reported outcomes (PROs) through web-based systems, including online self-reporting and monitoring of symptoms and health. Plenary speakers in this session shared their expertise and innovative projects on topics that cover each of these areas. This paper presents an overview of each of these presentations.

## Personal narrative: self-monitoring in Parkinson’s disease, Jon Stamford

E-health, with its emphasis on shared decision making and personal responsibility, is particularly well suited to use with chronic illnesses where patterns of health change may be both gradual and subtle. Small, slow improvements or declines in health, while largely imperceptible from day to day, can be readily unmasked by long-term monitoring.

Parkinson’s disease, a progressively neurodegenerative disorder of variable chronology [13], where periods of apparent stasis can be punctuated by stepwise increments in symptomatology, sits well within the scope of e-health. A typical clinical course with Parkinson’s disease may run for more than 20 years with appropriate management.

Diagnosis of Parkinson’s disease is usually made on the basis of three or four key motor symptoms—tremor, bradykinesia, rigidity, and postural instability—although the symptomatology of Parkinson’s disease also encompasses many non-motor symptoms. These non-motor symptoms include depression, anxiety, pain, constipation, anosmia, and sleep disorders among many others. Often these non-motor symptoms predate the clinical diagnosis of Parkinson’s disease, sometimes by as much as a decade. There is good evidence as well that non-motor symptoms play at least as large a part as motor symptoms in determining individuals’ quality of life [14].

**Fig. 1:**
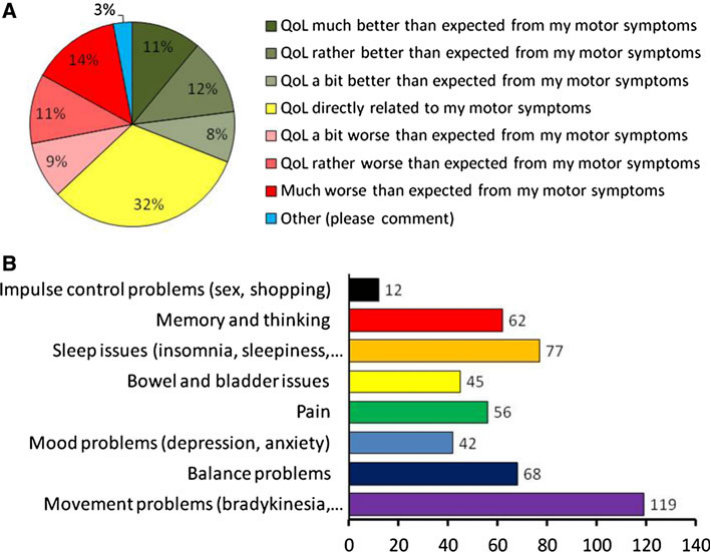
**a** Variability of quality of life in Parkinson’s patients. The percentage of patients categorized according to their relationship between quality of life and motor symptoms. **b** Prevalence of motor and non-motor symptoms reported to contribute to the quality of life. Data from 283 patients replying to a Parkinson’s Movement poll (October 2012)

Polls by Parkinson’s Movement have shown that, for patients, motor symptoms are a rather poor indicator of quality of life (Fig. 1a) and that non-motor symptoms are a major additional contributor (Fig. 1b). As with the motor symptoms, there is extreme variability in the numbers and extent of non-motor symptoms experienced by patients, presenting challenges for both treatment and assessment.

Parkinson’s disease presents a significant treatment challenge to neurologists for a number of reasons. Firstly, in seeing a patient only every several months, the resultant clinical picture is little more than a snapshot of a patient who may have overmedicated simply in order to make it to the clinic for assessment. Secondly, post hoc questionnaires of symptoms rely on accurate recollection by the patient and, in any case, are inevitably biased toward more recent events [15]. Thirdly, Parkinson’s disease has a highly variable clinical presentation. No two patients are alike in their combination of symptoms, making a “one size fits all” treatment algorithm unfeasible. Fourthly, even in the same patient, the day-to-day variability of the condition makes treatment generalization challenging [16]. Fifthly, clinical assessment of Parkinson’s disease seems historically to focus on motor symptoms. The importance of non-motor symptoms is only recently acknowledged. Sixthly, the health of most patients is dependent on compliance with a rigid medication schedule. Collectively, these considerations mean that treatment and assessment of Parkinson’s patients are highly individualized.

A positive response among the Parkinson’s community to this disheartening symptomatic individuality has been to take a strong personal interest in symptomatology and its relation to well-being. In consequence, a number of means of self-assessment have emerged. These range from simple charts and diaries to full-blown computer-based applications. While the former have the advantage of simplicity (and can be used in a power cut), the latter offers greater scope for data management and trend analysis. The ability to enter individual data into a centralized database offers the opportunity to contextualize one’s own patient experience. This is in many respects the model upon which *PatientsLikeMe* is predicated and which has proven engaging for so many.

However, ultimately neither personalized health diary nor large centralized database offers the perfect solution. Both scientists and patients have been increasingly engaged by the advent of wearable monitoring devices and the scope offered by these [17]. Of course for many patients, the most familiar “wearable” device is the mobile phone. Modern smart phones with gyroscopes, accelerometers, and processing power comparable to desktop computers have spawned a multitude of “apps” that, while not explicitly designed for use by Parkinson’s patients, have nonetheless proved popular. Among these are apps to measure reaction time, repetitive finger tapping, coordinated movement, and exercise parameters. Seismographic programs, although designed for earthquake detection, work extraordinarily well when used to record and quantify Parkinsonian tremor.

Although each of these applications has found favor among patients, they fall short of the ideal in four ways. Firstly, they address merely the motor symptoms of Parkinson’s disease. Secondly, they are not optimized specifically for the purposes for which they are being used. Thirdly, they lack the degree of interactivity and community, which adds value to their usage. Fourthly, and unsurprisingly, they lack the facility to give the patient reminders about taking their dose of anti-Parkinsonian medication, for instance.

The four considerations above are at least partially addressed by apps such as PD Life [18]. This app, although admirable in many ways, does not explicitly address motor symptoms (although these may be added). Apps are urgently required that combine physical measures of health with motor symptoms and patient perception of health. I know of at least 2 apps that are undergoing beta testing at the present time.

For many patients, personalized health monitoring is focused very much on the personal. Many patients are interested in no more than monitoring their own symptoms. They have no need to contextualize their experience. But for others, the opportunity to compare their data with the general population of patients with the same condition is an attractive feature. And many self-monitoring apps offer the facility to upload data to a central server.

This raises the question of data ownership. Perhaps as a result of earlier experiences, patients can be wary about entering large amounts of personal data into large centralized databases without concrete assurances of privacy. This is especially applicable to chronic health conditions such as Parkinson’s disease where details of health status may be perceived as being of interest to insurance companies for instance. Experience has taught that absolute transparency is essential to engage patient trust. On the whole, the database holders acknowledge individual ownership of individual data and ask patients for permission to use such data in anonymized form for other purposes. These might include the sale of de-identified data to the pharmaceutical or insurance companies, but, more commonly, the data are used for research.

Returning finally to my initial theme and the suitability of Parkinson’s disease as a test vehicle for self-monitoring, it is apparent that the condition presents both opportunity and challenge. The principal opportunity lies in the range of symptoms that may/should be monitored and the many correlations and associations with quality of life that this engenders. For the patient who is engaged with his/her condition, there is scope for the patient to make a tangible contribution to shared decision making. The principal challenge for any new app is the extent of engagement with the Parkinson’s community. One of the less widely reported non-motor symptoms is apathy, essentially an early step on the path to depression. Apathy can mitigate the best-intentioned research and has strong implications for health-related quality of life. The challenge for the most engaged patients with Parkinson’s disease will be to communicate that enthusiasm and engagement to those who are not enthused or engaged. Innovations in e-health will be most meaningful when they are universal.

## Wider perspective: online social networks and emerging technologies, Paul Wicks

In the past 5 years, a vanguard of patients with serious, life-changing illnesses such as Jon have used technology to connect with one another, participate in research, and better advocate for themselves as empowered patients. But how credible is this movement?

In the early 2000s, patients with neurological conditions including Parkinson’s disease communicated with one another through hosted communities such as BrainTalk, based at Massachusetts General Hospital (MGH) [19] or BUILD (for amyotrophic lateral sclerosis (ALS) patients) at King’s College Hospital [20]. The advantage of having a reputable organization like MGH or King’s involved was that patients felt they could trust the site, clinicians would feel comfortable referring patients there, and the insights generated could be reflected back into the scientific literature by researchers. There were disadvantages too, however; if the site were to crash, there were no backup sites where patients could fall back to. To maintain their reputation, such communities had a strict set of policies governing patient-to-patient interactions which some felt draconian or inhibitory to conversation. By the mid 2000’s, advances in Internet technology meant services such as Yahoo Groups or Ning allowed the rapid formation of new networks that could be patient led at minimal cost and with their own rules. However, this diffusion of expertise could itself be a limitation; Was it better to have dozens of fragmented online communities for people with Parkinson’s disease all over the web? Or did this lead to more duplication and wasted effort?

More recently, there have been an explosion in interesting online communities [21, 22], but here, we will explore two that combine aspects of social networking with credible scientific and quantifiable data collection; PatientsLikeMe and 23andMe.

PatientsLikeMe was founded by a family affected by ALS to enable patients to record their disease progression using clinically validated outcome measures [23], share their experiences with others at an individual [24] and aggregate level [25], and eventually to further the course of research by crowd-sourcing information on what works and what does not [26]. To date, researchers on the site have produced over thirty peer-reviewed publications: from a clinical trial conducted over the Internet [26] to the development of new patient-reported outcomes in ALS [27] and multiple sclerosis [28], the detection of medication side effects and disease variability in Parkinson’s disease [16, 29], new measures of treatment adherence [30], and even biologically driven studies exploring the pathogenesis of disease [31]. Clinically, the site has been controversial because telling patients how sick they are and how they fit in a wider context with regard to their progression or options has traditionally been viewed as the role of their physician. From a research perspective, the site attracts a self-selecting and therefore somewhat biased subset of patients whose biases vary by disease, but for instance in the case of multiple sclerosis skew a little younger and more likely to be female than neurological patients seen at a specialist clinic [32].

A second innovative platform is 23andMe, an online community that relies less on quantified self-report in patients and more on genetic data extracted from saliva samples collected by mail and analyzed for single nucleotide polymorphisms (SNPs) using regularly improving techniques. Once the SNPs are analyzed, customers receive access to a Web site detailing what their results mean and providing their predicted risks of disease like Alzheimer’s or psoriasis, as well as non-health-related tools such as ancestry mapping. Like PatientsLikeMe, there is a forum for discussing findings, though the sharing of data is more carefully controlled; members of 23andMe can invite one another to “share” their results, though they choose to restrict this sharing to only a basic SNP dataset which excludes the most serious health traits.

Although initially criticized by some bioethicists for providing patients with ambiguous or potentially alarming results without the support of a traditional genetic counselor, 23andMe is interesting because it disintermediates mediates traditional barriers to letting patients try to understand their own health risks. They too have sought to produce useful and validated research findings to demonstrate their commitment to science [33], presenting as many as 16 posters at the American Society of Human Genetics meeting in 2012, for instance (http://blog.23andme.com/23andme-research/23andmes-presentations-at-ashg/).

It would be an understatement to say both sites have been controversial; 23andMe in particular has provided ample opportunity for academics to generate papers about the potential ethical pitfalls of direct-to-consumer genetic testing. But as they are popular with their users, continue to operate sustainable business models, and collaborate widely with bona fide academic institutions, they are likely to force reconsideration of existing principles of research.

Both online platforms use PROs as a tool for self-monitoring and research, and although these can be developed, validated, and refined to high standards, they remain an imperfect means of measurement. Here is where insights from the non-medical “consumer health” space are integrating into medicine. For example, if you were trying to keep an accurate measure of your running distances and speeds, you could try and self-report how fast you were going or time yourself with a watch. But with a smartphone and freely available applications (“apps”) such as Run Keeper, the data can be gather objectively and much more accurately through embedded GPS, accelerometer, and Internet connectivity. Through social portals and connections to social networks, we can even add motivational overlays, such as having friends give us encouragement to be compliant to our running schedule or congratulating us for running a personal best. What happens when we integrate tools like this into online medicine?

Three major trends appear tantalizing. First, sensors are everywhere, they are getting better, and they are getting cheaper. Earlier in the decade, the only reliable way to capture quantitative data about walking in a disease like Parkinson’s disease would be a complicated arrangement such as motion capture system [34], whereas now it is possible to use off-the-shelf technology such as an Xbox Kinect^®^ [35], which allows home use cheaply, albeit with reduced resolution. Looking forward, wearable computing technologies such as Google Glass could allow for real-time passive capture in ecologically valid situations. Glass includes an accelerometer, GPS, still camera, video camera, and connection to a smart phone—so for example, in assessing someone with Parkinson’s disease, it should be possible to naturalistically record instances where activities of daily living such as making a hot drink have been impaired by tremor. These could be archived for physician review or even quantified to measure the effects of treatment.

The second important trend is telemedicine through web-enabled video cameras (“webcams”), which has the potential to broaden specialist access to a much wider pool of patients. For example, the specialist movement disorders team at Johns Hopkins is able to remotely care and advise hundreds of patients with Parkinson’s disease in a nursing home many miles away [36]. Care remains local, but expertise is something that transmits easily. In a small randomized control trial, Dorsey’s group found that telemedicine visits saved the average patient 100 miles of travel and 3 h of time and 85 % of participants said they would rather conduct further visits remotely than in person [37].

Finally the “Internet of Things” [38] takes these elements even further, through the use of low-cost disposable sensors such as RFID chips which could have implications for tracking observations of daily living (e.g. fall sensors in clothing, usage sensors in wheelchairs, soiling sensors in continence pads, walking sensors in shoes), connectivity for every day objects (e.g. a car that assesses your driving ability in real time), or specific medical applications (e.g. medication compliance, remote calibration of a deep-brain stimulator, interaction between food or utensils and insulin pumps).

The exciting challenge for us in quality of life research is to think what clarity we could bring to those aspects of human life that remain imperceptible and personal after all these technologies have been brought to bear. Can we be as rigorous about the internal world as these tools will allow us to be about the physical one?

## Integrating patient-reported outcomes in pediatric clinical practice using a web-based tool, MA Grootenhuis and L Haverman

In the past 10 years, there has been a growing interest in the use of health-related quality of life (HRQOL) PROs in clinical practice. Research in adult patients shows that the integration of PROs in clinical practice generally improves patient–clinician communication; PROs help in identifying and discussing HRQOL issues and add to improvement of a patient’s health outcomes and satisfaction with care [39, 40]. PatientViewpoint, for example, is a Web site that collects PROs in outpatient clinical oncology and links the data with the patient’s electronic medical record. Initial results indicate that such a system could improve the quality of cancer care [41]. Similarly, the use of touch-screen completion of HRQOL with feedback to physicians was found to improve patient–provider communication and patient well-being [42].

Studies on the use of PROs in pediatric clinical practice were scarce compared to adult practice [43] while there is a particular need to address HRQOL in daily pediatric clinical practice. As a result of the improvements in medical care, the prevalence of chronic illness in children has increased worldwide. At least 14 % of children grow up with a chronic illness [44]. In the context of a child’s development, the repeated measurement of HRQOL in different developmental stages can be a valuable addition to the clinical consultation.

In 2005, the psychosocial department of the Emma Children Hospital started with a study on the use of PROs in pediatrics: The Quality of Life in Childhood Oncology (QLIC-ON) study. During the QLIC-ON study, HRQOL questionnaires were completed at the clinic immediately before the actual doctor’s visit, with patients using stand-alone computers. A printed version of the PRO (so-called PROfile) was handed to the pediatrician to be discussed during the consultation. Engelen et al. [45] showed that the feedback of HRQOL via PROs during the consultation with the pediatric oncologist increased the discussion on emotional and psychosocial functioning and improved the identification of emotional problems in pediatric oncology patients. In addition, the intervention did not lengthen the duration of the consultation [46]. The method to gain the PROs and to provide the PROfile to the pediatric oncologist was very time-consuming and often caused logistical problems because of lack of privacy and room at the clinic. It was concluded that the use of a web-based program could overcome these problems and could contribute to an improvement in the use of PROs in clinical practice [47].

To study the use of electronic PROS (ePROs) in clinical practice using a Web site, we conducted a new multicenter study, the KLIK study (in Dutch: Kwaliteit van Leven In Kaart, in English: Quality of Life in Clinical Practice). We developed a Web site (http://www.hetklikt.nu), and children between 0 and 18 years old with juvenile idiopathic arthritis (JIA) were included. Children and/or their parents completed the HRQOL questionnaires on the Web site at home. The answers on the questionnaires were schematically converted into a so-called KLIK “ePROfile.” Pediatric rheumatologists could retrieve these ePROfiles directly from the Web site during the consultation. The web-based ePROfile appears to be an efficient application to systematically pay direct attention to HRQOL issues in daily pediatric clinical practice. Our study shows that providing information on patient’s HRQOL to the pediatric rheumatologists leads to significantly more discussion of emotional and social functioning during consultation and improves the pediatric rheumatologists’ satisfaction with the provided care. Overall, parents, children, and pediatric rheumatologists evaluate the use of the ePROfile positively [48].

Our hospital appeared to be ready to incorporate systematic attention for HRQOL in pediatric clinical practice as a result of the positive findings in both adult and pediatric care. Therefore, we decided to implement the use of the KLIK ePROfile in daily clinical practice for children with various chronic illnesses. We started with the KLIK implementation in our hospital in June 2011, and now in January 2013, 13 pediatric patient groups have started with the use of KLIK in daily clinical practice. So far, more than 100 professionals followed the training course and over 1200 patients are registered on the KLIK Web site of the Emma Children’s Hospital. Apart from PROs, we have also added parent-reported outcomes (ParROs) to the KLIK web portal.

Besides the implementation in our hospital, we also started to implement KLIK in other hospitals and clinics, as a result of collaborative projects. In the upcoming years, we will continue to implement KLIK in more pediatric patient groups. Pediatricians working with the KLIK ePROfile recognize the importance of monitoring HRQOL and tell their colleagues, which encourage others’ interest to use KLIK in the near future. Besides including more patient groups, we also want to start using KLIK for children in transition to adult care. Transition of adolescents from pediatric to adult care can be challenging [49]. As one part of the solution, KLIK can be used as a tool to help fill the gap between the pediatric and adult health care for adolescents. For example, KLIK can be adapted to help to convey needed information between the pediatric and adult healthcare teams during the transition process.

The implementation of the use of PROs in daily clinical practice is a continuous process and creates new challenges and opportunities for care, as is extensively described in the International Society for Quality of Life Research (ISOQOL) guidelines [50, 51]. We have described the KLIK implementation following these methodological recommendations composed by the International Society for Quality of Life (ISOQOL) [52].

## Conclusions and future directions

Each of the presentations in this session conveyed a complementary perspective on the use of e-health technologies and their potential benefits and challenges. Advances in technology are generating new opportunities to leverage e-health tools to help individuals self-monitor and assess their symptoms and health, create online communities, and incorporate the routine collection of PROs in clinical practice. The common thread across all the talks was the application of e-health tools to empower individuals with chronic disease to be actively engaged in the management of their health.

With the potential benefits that may be derived from e-health technologies come potential challenges that have likely contributed to the slow adoption of e-health in routine care. There is no one size fits all solution, and matching the right technology for a given patient population or desired clinical objective is key to ensuring sufficient perceived usefulness and uptake. Combining different solutions and applications such as personal health records with home monitoring devices and sensors, and social networking will help to tailor new technologies to individuals preferred method of managing their health. Interaction between broad social networking and personal clinical care will also require a shift in the way health professionals collaborate with patients.

Privacy data protection and ownership is a major concern of any Internet-based application. The balance between the patient as the owner of data and the medical and academic profession’s documentation and use of the data must be struck, with patient confidentiality always at the forefront without impeding the development of innovative solutions.

The vast amount of information online, the majority of which is not reviewed by a credible organization, makes it difficult for patients to effectively select information that is valid and dismiss non-validated or potentially harmful information. Technology that incorporates information filters and decision support can help match relevant information to the individuals’ preferences, clinical profile, and social and enviornmental context.

Making e-health universal, and not limiting its application to a self-selecting demographic profile will also be important. Equitable health care is one of the promises of e-health, but at the same time, there is a considerable threat that e-health may create a digital divide and deepen the gap between the people, who do not have the skills, and access to computers and networks, and those who do. The patient population sub-groups who do not have the skills and access are likely to benefit most from health technologies. Surveys have shown that computer use is lower among groups with lower incomes, lower education, blacks, and those over 60 years old. Hospitals serving a greater proportion of low-income patients were even less likely to adopt electronic medical records and those without digital systems tended to have an inferior quality of care [53]. Policy and political will be needed to ensure equitable access for all. Early promising mechanisms have been instigated including providing access to computers and Internet in schools, and governments providing extra support to clinical settings in low-income areas to implement electronic health records [53]. Ongoing implementation and monitoring of such efforts will be important for ensuring that countries continue to work toward equitable access for all.
